# Müllerian Remnant Cyst as a Cause of Acute Abdomen in a Female Patient with Müllerian Agenesis: Radiologic and Pathologic Findings

**DOI:** 10.1155/2016/6581387

**Published:** 2016-06-30

**Authors:** Mujtaba Mohammed, Mary Allen-Proctor, Andrij Wojtowycz

**Affiliations:** ^1^Department of Radiology, SUNY Upstate University Hospital, Syracuse, NY 13210, USA; ^2^Department of Pathology, SUNY Upstate University Hospital, Syracuse, NY 13210, USA

## Abstract

We report a case of a 17-year-old female with Müllerian agenesis who presented with right sided abdominal pain clinically suspicious for acute appendicitis. Multimodality imaging workup revealed a heterogeneous cystic right upper quadrant mass with surrounding fluid and inflammatory changes. Surgical resection of this mass was performed and a histopathologic diagnosis of a hemorrhagic Müllerian remnant cyst was made, which to the best of our knowledge has never been described in a patient with Müllerian agenesis.

## 1. Introduction

Müllerian agenesis, also referred to as Mayer-Rokitansky-Kuster-Hauser (MRKH) syndrome, is a rare congenital abnormality that occurs in females and primarily affects the reproductive system. It is characterized by agenesis or underdevelopment of the uterus and vagina with normal development of the ovaries and secondary sexual characteristics [1]. Abnormalities of the reproductive system increase their predisposition to unusual causes of abdominal pain such as hematocolpos/hematometra. There is also increased incidence of ovarian maldescent in females with Müllerian duct anomalies who may present with atypical abdominal pain when ovaries are located within the abdomen [2]. We describe an unusual case of acute abdomen in a patient with Müllerian agenesis.

## 2. Case Presentation

A 17-year-old female presented with acute right sided abdominal pain that had been progressively getting worse for the previous 3 days. Her past medical history was significant for Müllerian agenesis and lack of a uterus. Her past surgical history was significant for small bowel atresia and resection of a portion of her small bowel during infancy. Physical examination revealed tenderness to palpation in the right lower quadrant and the periumbilical region. Laboratory tests were significant for leukocytosis with a left shift and an elevation of C-reactive protein.

**Figure 1:**
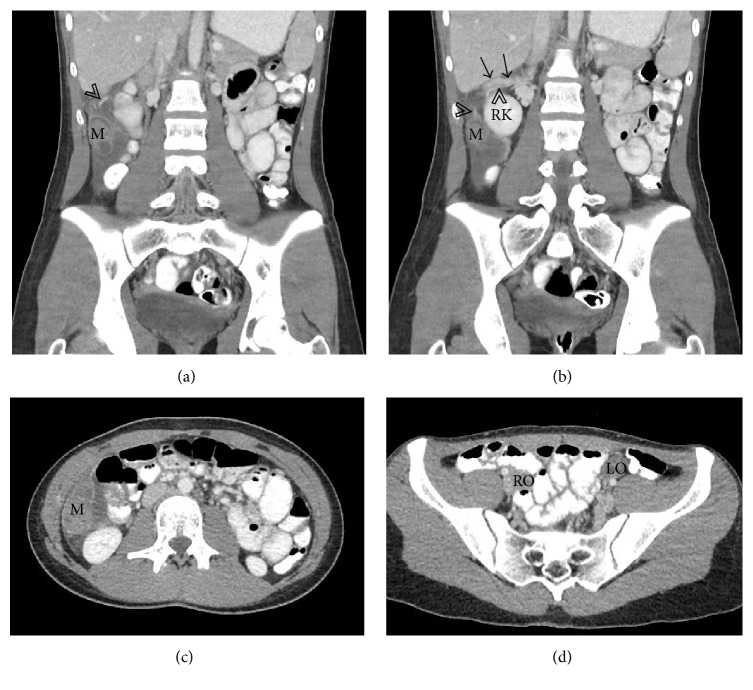
Images (a), (b), and (c) demonstrate a heterogeneous structure (M) in the right upper quadrant with its supplying artery (arrowheads) and venous drainage (arrows) adjacent to the right kidney (RK). Axial CT image (d) shows both the right (RO) and left (LO) ovaries in the pelvis.

**Figure 2:**
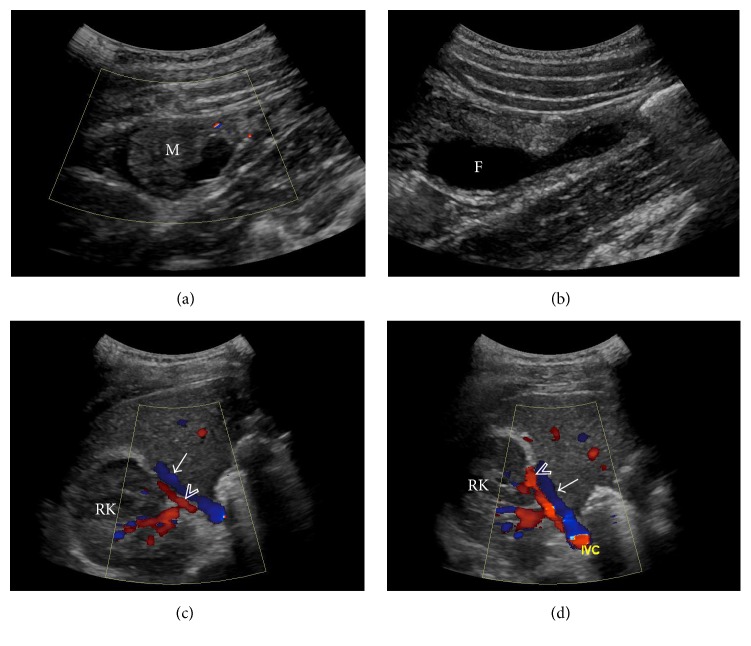
Image (a) is a sagittal sonographic image of the right upper quadrant demonstrating a structure (M) with no internal flow on color Doppler. Fluid (F) is seen just below the mass on image (b). Axial sonographic images (c) and (d) demonstrate the supplying artery (arrowhead) and the venous drainage (arrow) adjacent to the right kidney (RK).

**Figure 3:**
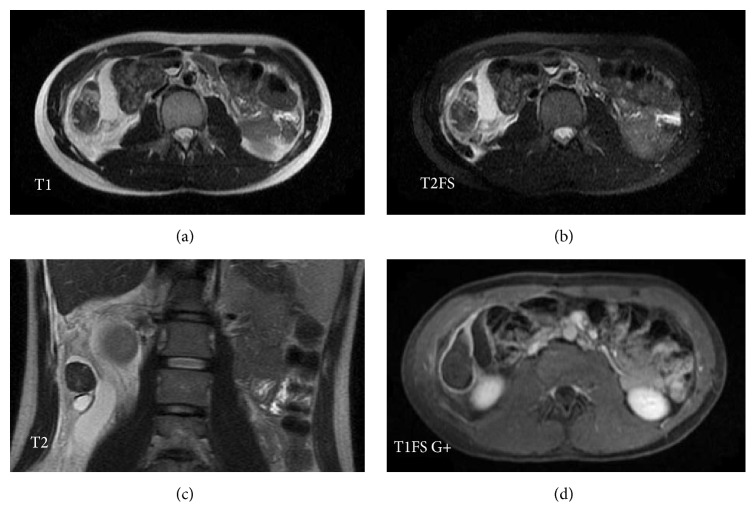
Axial MRI images (a) and (b) demonstrate a mostly hypointense structure on T1 and T2FS sequences in the right upper quadrant with surrounding fluid and edema. On the coronal T2 image (c), a cystic component is seen inferior to this mostly T2 hypointense mass. Axial post contrast image (d) shows peripheral enhancement.

CT scan of the abdomen and pelvis demonstrated a heterogeneous structure measuring 3.8 × 2.2 × 2.6 cm (transverse × anteroposterior × craniocaudal) inferior to the right hepatic lobe and posterolateral to the ascending colon with blood supply from a branch arising off the right renal artery and a draining vessel into the inferior vena cava (Figure 1). A low density cystic component was seen inferiorly within the mass. There was a small amount of surrounding free fluid and fat stranding. The uterus was absent and both ovaries were present in the pelvis. Ultrasound exam (Figure 2) of the right upper quadrant revealed a heterogeneous mass with no internal blood flow and confirmed the arterial and venous connections noted on the CT scan. MRI exam of the abdomen (Figure 3) showed a mass with intermediate to low signal intensity on T1 and a low signal intensity on T2 with a cystic component inferiorly. Extensive surrounding edema and rim enhancement were also present.

**Figure 4:**
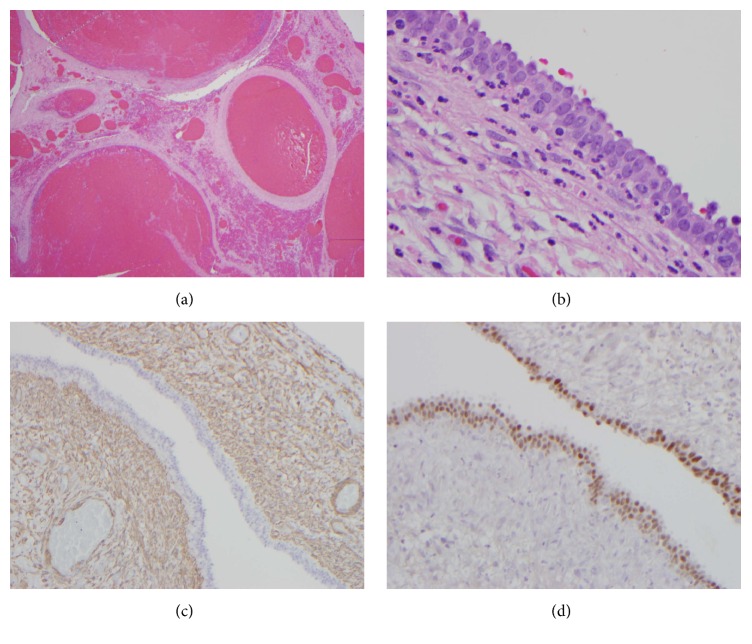
Histopathological images of the resected specimen. Image (a) demonstrates extensive hemorrhagic soft tissue (low power, H&E). Image (b) shows cyst wall lined by cuboidal epithelium (high power, H&E). Image (c) demonstrates smooth muscle in the cyst wall after immunohistochemical staining with smooth muscle actin (medium power, SMA). Image (d) demonstrates positive nuclear immunostaining with PAX-8 (medium power, PAX-8).

The patient underwent laparoscopic surgery for a presumptive diagnosis of a possible torsed supernumerary ovary or acute appendicitis with perforation. The resected gross specimen consisted predominantly of yellow lobulated adipose tissue and a portion of hemorrhagic tubulocystic structure. Microscopic examination demonstrated mature adipose tissue and abundant hemorrhagic tissues (Figure 4(a)). The wall of the cystic structure was lined by cuboidal epithelium (Figure 4(b)). Immunohistochemistry for PAX-8 and smooth muscle actin (SMA) showed the presence of Müllerian type epithelium and smooth muscle within the cyst (Figures 4(c) and 4(d)). The hemorrhagic soft tissue showed no evidence of follicles. The histopathological diagnosis was consistent with Müllerian derived remnant cyst with extensive hemorrhage.

## 3. Discussion

Müllerian cysts in females are usually seen within the pelvis and typically mimic cysts of ovarian origin [3]. Müllerian cysts of the upper abdomen are an extremely rare occurrence in females [4] and have never previously been reported in a patient with Müllerian agenesis. Histologically, the cysts have a smooth muscle wall and are lined with either cuboidal or columnar epithelial cells. The histogenesis of the development of these cysts is unclear with several proposed theories of origin including caudal growth of the developing mesonephric duct, ectopic ovarian tissue, a secondary Müllerian system, and endometriosis [3, 5]. The differential considerations in this adolescent female presenting with abdominal pain would most commonly include appendiceal or ovarian pathology as well as other unusual causes such as hematocolpos/hematometra and ovarian maldescent, especially if there is known history of Müllerian agenesis. Imaging findings of an upper abdominal heterogeneous cystic mass with distinct arterial supply and venous drainage, in a patient with Müllerian agenesis, should raise the possibility of a Müllerian remnant cyst.
